# Humans as holobionts: implications for prevention and therapy

**DOI:** 10.1186/s40168-018-0466-8

**Published:** 2018-05-01

**Authors:** Maarten van de Guchte, Hervé M. Blottière, Joël Doré

**Affiliations:** 1 Micalis Institute, INRA, AgroParisTech, Université Paris-Saclay, 78350 Jouy-en-Josas, France; 2 MetaGenoPolis, INRA, Université Paris-Saclay, 78350 Jouy-en-Josas, France

## Abstract

The human gut microbiota is increasingly recognized for its important or even decisive role in health. As it becomes clear that microbiota and host mutually affect and depend on each other in an intimate relationship, a holistic view of the gut microbiota–host association imposes itself. Ideally, a stable state of equilibrium, homeostasis, is maintained and serves health, but signs are that perturbation of this equilibrium beyond the limits of resilience can propel the system into an alternative stable state, a pre-disease state, more susceptible to the development of chronic diseases. The microbiota–host equilibrium of a large and growing proportion of individuals in Western society may represent such a pre-disease state and explain the explosive development of chronic diseases such as inflammatory bowel disease, obesity, and other inflammatory diseases. These diseases themselves represent other alternative stable states again and are therefore hard to cure. The holistic view of the microbiota–host association where feedback loops between microbiota and host are thought to maintain the system in a stable state—be it a healthy, pre-disease, or disease state—implies that integrated approaches, addressing host processes and microbiota, should be used to treat or prevent (pre-)disease.

## Background

Research on the human gut microbiota and its importance for health has come a long way since the beginning of the twentieth century, when Metchnikoff suggested that the live bacteria in yogurt exerted health-beneficial effects on the consumer, beyond the earlier recognized implication of (commensal) gut bacteria in food digestion and pathogen exclusion [1]. This concept of what we now call probiotic effects led to the development of a large body of research documenting the existence of direct interactions between non-pathogenic, transiting, or commensal, gut bacteria and the host, implicating dedicated host cell receptors and signaling pathways. Gut bacteria turned out to influence fundamental host processes including metabolism, adiposity, maturation, and modulation of the immune system and even brain function and decision making [2–6]. The host in turn creates the conditions that support, allow, or inhibit the development of specific (groups of) bacteria and responds to signals emitted by the microbiota.

In parallel, the development of high-throughput DNA sequencing techniques yielded access to the quantitative composition of an individual’s dominant gut microbiota, including the estimated 70% or so of uncultured bacteria, at the species (or OTU) level or at the gene level [7, 8]. These techniques for the first time allowed the characterization and comparison of the microbiota of large cohorts of healthy subjects and patients to an unprecedented level of detail and provided the statistical power to reveal different microbiota types in healthy subjects and atypical microbiota compositions in patients for a growing number of diseases [9]. Going beyond correlation, experiments in mice established a causal role for the gut microbiota in obesity, through the transfer of an obese phenotype from obese to germ-free non-obese mice by fecal transplantation [10, 11]. Similar results have since been reported with regard to inflammatory bowel disease (IBD) [12] and the propensity to develop non-alcoholic fatty liver disease [13] or alcoholic liver disease [14] in mice, or depression in rats [15], in the latter two cases after fecal transplantation from human donors.

Thus, a general picture emerged of an intimate relationship between humans and their gut microbiota, where both rely on each other to maintain a stable state of homeostasis that can be called “health.” In view of the accumulating evidence of the profoundness of this relationship and its significance for health, humans and their gut microbiota can be considered as holobionts (in the sense that the fitness of the host depends on and cannot be seen separate from its microbiota [16, 17]). Although departing from the original definition of the term holobiont, this interpretation is increasingly used to describe the host–microbiota relationship (see references [18–20] for review and discussion). While the microbiota of other body sites (oral cavity, vagina, airways, skin) can be included in this holobiont view and the ideas we expose here, we limit our discussion to the microbiota of the gastro-intestinal tract as its interplay with systemic health is the most extensively documented. Full acknowledgment of the holobiont condition will have important consequences for the definition of health-nutrition strategies for disease prevention and translational research for the therapeutic treatment of diseases.

## Main text

### Non-random gut microbiota assemblies

Recent studies of human gut microbiota composition revealed a number of noteworthy characteristics of this microbial community now thought to play an extremely important, or even decisive, role in health. Only a limited number of the known bacterial phyla are represented among the dominant gut microbiota (mainly Bacteroidetes and Firmicutes, and to a lesser extent Proteobacteria and Actinobacteria [21]). When comparing the gut microbiota of large numbers of individuals, it appears that community assemblies are not random or equally distributed. Thus, two or three so-called enterotypes [22] have been described, each named after a “driver” genus (*Bacteroides*, *Prevotella*, and, in some studies, *Ruminococcus*) [22, 23]. Human enterotypes have been associated with long-term dietary habits, where the *Prevotella* enterotype appears to be preferentially associated with a high-fiber diet, enriched in fruits and vegetables, while the *Bacteroides* enterotype appears to be linked to a higher consumption of animal fat and proteins [23]. Although relatively stable and resistant to short-term dietary intervention [23], enterotypes are not strictly separated entities and the microbiota of an individual may (temporarily) switch from one enterotype to another [24]. Although the concept of enterotypes has been much discussed, differing views have at least in part been reconciled to recognize the reality of configurations of relative microbial abundance that occur more frequently than others [25]. Some have preferred to take the ratio of *Prevotella*/(*Bacteroides* + *Prevotella*) as an indicator of microbiota type, which reveals clear bimodal distributions across several studies [26].

At another level, when classifying human gut microbiota samples by “gene richness”—a measure of microbiota diversity that counts the number of different bacterial genes in a sample—a clear bimodal distribution is observed with microbiota having either a “low gene count” (LGC) or a “high gene count” (HGC) [27]. Enterotypes and gene richness categories overlap to a large extent, with the LGC or low bacterial diversity group roughly corresponding to the *Bacteroides* enterotype, while the HGC or high bacterial diversity group more or less corresponds to the *Prevotella* (and *Ruminococcus*) enterotype (E. Le Chatelier, personal communication). Of interest, obese individuals with low gut bacterial diversity are characterized by more marked overall adiposity, insulin resistance and dyslipidemia, and a more pronounced inflammatory phenotype, when compared with individuals with a more diverse microbiota [27], and the former may be more prone to develop inflammation-related cardiometabolic comorbidities.

Apart from these general overarching classes of human gut microbiota, atypical microbiota compositions are observed in an ever-growing number of diseases. In the case of obesity, the correlation has proven so strong that microbiota composition becomes a powerful diagnostic tool, outperforming traditional human biomarkers of disease (receiver operating characteristic (ROC) analysis of 9 signature microbiota species vs ROC analysis of 32 human genome loci associated with adiposity measures) [27]. For Crohn’s disease and cirrhosis, Microbiota Dysbiosis Indices have been proposed that correlate with disease status [28, 29]. For some diseases, like obesity, insulin resistance, IBD, depression, and the liver diseases cited above, the association has gone beyond correlation, with causal relationships established in animal models.

### Alternative stable states of the gut microbiota–host symbiosis

It thus becomes clear that discrete states can be recognized in adult human gut microbiota composition that are much more frequently encountered than intermediate compositions. Atypical discrete states are increasingly linked to different states of human health, be it different overt diseases or an ensemble of physiological and immunological parameter values that may indicate a propensity to disease development, a “pre-disease state,” as may be the case in LGC individuals. A pre-disease state may also be suspected in carriers of a *Bacteroides* enterotype microbiota, which was found to be associated with increased lymphocyte counts and C-reactive protein levels [25]. Discrete states in gut microbiota composition therefore appear to indicate differences in ecosystem function, or ecosystem services, of the microbiota–host symbiosis. Microbiota gene function analyses support this idea, as exemplified by functional shifts in the LGC microbiome where functions related to mucus degradation and oxygen tolerance or oxidative stress responses are overrepresented, while functions involved in butyrate production are underrepresented, compared to HGC microbiota [27]. This notion is coherent with the succession of different microbiota compositions through the different phases of life, where the microbiota is thought to fulfill different functions in each phase (reviewed in [30]). The microbiota compositions associated with chronic inflammatory diseases or with a pre-disease state are atypical, however, and not part of the habitual succession of compositions.

**Fig. 1:**
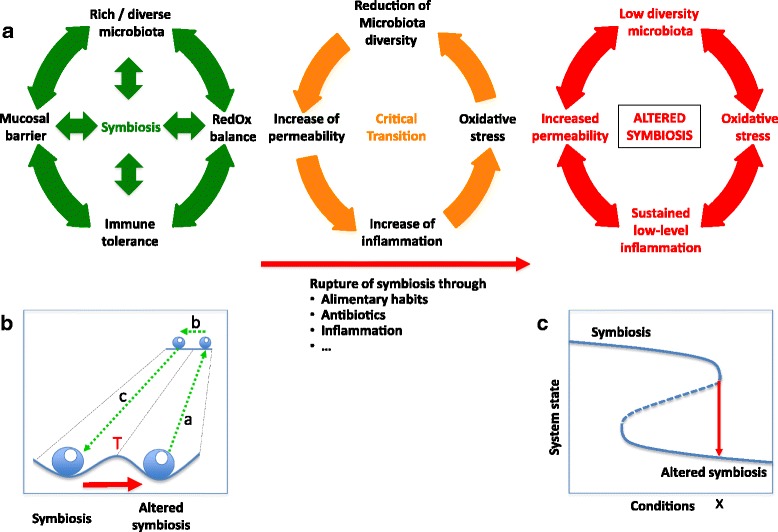
Alternative stable states and critical transition in the gut microbiota–host symbiosis. **a** Alternative stable states representing health (symbiosis) (left) or (pre-)disease (altered symbiosis) (right). The cycle in the middle represents a vicious circle of self-enhancing deterioration of symbiosis, leading to critical transition to an alternative stable state of altered symbiosis. Changing alimentary habits could start this self-enhancing process through reduction of microbiota diversity or by increasing permeability of the mucosal barrier. Antibiotics can do the same through reduction of microbiota diversity. **b** Schematic representation of alternative stable states as beads in a landscape. T indicates a tipping point. In case of (pre-)disease (altered symbiosis), symbiosis may be restored through dual action via (a), (b), and (c) (see text for explanation). **c** Alternative stable states (top and bottom part of the curve) are different states that can exist under identical external conditions. The dashed line represents a tipping point (cf panel **b**: T). When the conditions change to a point beyond x, the system will switch to the alternative stable state (altered symbiosis). If the starting point (symbiosis) is situated in the bi-stable range of conditions, setting back the conditions to those that reigned before the switch to the state of altered symbiosis is not sufficient to shift the system back to its original state of symbiosis

The observation of discrete states in the usually stable adult human gut microbiota very much resembles what can be observed in many other (eco)systems [31, 32]. A well-known concept in ecology describes so-called alternative stable states as different attraction points in the space of theoretical possibilities, under given conditions. Schematically, alternative stable states are often represented as beads in the valleys of a landscape (Fig. 1b). From this representation, it is intuitively clear that when a system is pushed to its limits (by stochastic movements, perturbations, changing conditions, or a combination of these factors), it can reach a “tipping point” (Fig. 1b) from where it can easily be propelled to a different state. Such a state transition is also called a “critical transition” or a “catastrophic transition,” meaning that setting back the conditions to those that reigned before the switch is not sufficient to shift the system back to its original state (Fig. 1c). The original and the new state are thus alternative stable states, different stable states that can exist under the same external conditions (Fig. 1c) [33].

Accumulating evidence suggests that various factors often referred to as “Western lifestyle” elements may lead, or already have led, to critical transitions in the adult human gut microbiota–host ecosystem. The earlier mentioned link between long-term diet and enterotype is a clear example of the influence of changing conditions, as the study by Wu et al. [23] strongly suggests that the *Prevotella* enterotype is an ancient enterotype (linked to “ancient” dietary habits), that long-term Western dietary habits have led to a switch to the *Bacteroides* enterotype, and that reversal cannot be accomplished by a short-term return to a low-fat/high-fiber diet. Mechanistic insight in how fiber deprivation can lead to degradation of the colonic mucus barrier, low-level inflammation, and enhanced pathogen susceptibility [34] further supports the idea that Western dietary habits may cause a critical transition of the bacteria–host symbiosis to a pre-disease state.

A second example of how Western dietary habits can induce shifts in the gut ecosystem is provided by the effects of dietary emulsifiers, detergent-like molecules that are a ubiquitous component of processed foods. In mice, emulsifiers can induce a reduction in gut microbiota diversity, erosion of the protective function of the mucus, increased gut permeability, and low-grade inflammation and metabolic syndrome [35]. Transfer of the altered microbiota conferred low-grade inflammation and increased adiposity and dysglycemia to germ-free mice.

The Western lifestyle also brings temporal perturbations of the gut ecosystem which may bring the system close to and beyond a tipping point and thus have important long-term consequences in the form of a pre-disease state. Indications are getting stronger that antibiotics can have such an effect. The (over)use of antibiotics, especially in early life, can cause long-lasting reduction of gut microbiota diversity [36] and low-level inflammation [37] and is associated with an increased risk for a variety of diseases including obesity, types 1 and 2 diabetes, IBD, celiac disease, allergies, and asthma [37, 38].

The latter example illustrates the importance and vulnerability of the early stages of life, where both the microbiota and the host undergo important interdependent changes in a process of maturation preparing for adult life [30]. Vertical transfer of microbiota from mother to child in the perinatal period sets the basis for future developments, as becomes clear from the lasting effects of delivery mode (vaginal vs C-section) on microbiota composition [39]. This may mean that a (pre-)disease state microbiota of the mother can predestine the child to developing a (pre-)disease state, and there are indications that this is what happens [40]. Of note, while these characteristics of the developing intestinal ecosystem in early childhood may contribute to the propagation of a pre-disease state, they may also represent a window of opportunity for preventive action.

### From pre-disease state to disease. And back?

The Western lifestyle influences have in common that they do not immediately lead to overt disease. They do however change microbiota composition and (local) inflammatory status of the host, two conditions that can mutually sustain each other and propel the system to an alternative stable state (Fig. 1a). Growing epidemiological and experimental evidence suggests that such an alternative state indeed exists and can be regarded as a pre-disease state, with an increased susceptibility for overt disease development. The intensification and spread of Western lifestyle influences, possibly amplified by the mother to child transfer of microbiota and lifestyle habits, lead to a growing number of individuals in such a pre-disease state. From there, depending on host genotype and additional perturbations, the system can evolve to various other alternative stable states representing overt chronic diseases, each characterized by specific microbiota and host parameters.

This sequence of events could be (part of) the explanation for the strong, sometimes exponential, rise in the incidence of chronic inflammatory diseases over the last 60 years [41]. These diseases by their very nature show that the restoration of a healthy gut microbiota–host symbiosis can be a real challenge, a hallmark sign of critical transition. IBD, notably, can get to a point where there is no other option than the surgical ablation of the inflamed part of the intestine. And even then, relapse is regularly observed (> 50% within 1 year for Crohn’s disease).

In the holistic view of humans and their gut microbiota, a patient’s gut ecosystem represents an alternative stable state where microbiota and host mutually sustain each other in a condition of altered symbiosis (Fig. 1a). Therapeutic approaches should aim at breaking this condition of self-maintained disease through simultaneous action on different aspects, rather than the often ineffective current approaches that target the symptoms (i.e., inflammation). Microbiota and host should be in tune, implying that only correcting the one or the other may not work (and clinical experience shows that often it does not work). A combined action approach should target the microbiota (via diet, probiotics, or microbiota complementation or restoration) and diverse aspects of inflammation (gut permeability, inflammation per se, and ensuing oxidative stress). Coming back to the metaphor of the beads in a landscape depicting alternative stable states (Fig. 1b), cure may pass through a combination of actions that push the system back to the original state and actions that flatten the landscape or move the system to a flatter landscape, to facilitate backward transition. This is illustrated in Fig. 1b by the arrows (a), (b), and (c). Arrow (a) may for example represent a temporary treatment with anti-inflammatory, anti-oxidant, and/or other drugs. Arrow (b) may represent microbiota therapy and/or diet control, during the same period as treatment (a). Treatment (a) may subsequently be terminated, while keeping control of diet, to move the system back to a healthy state of symbiosis, as depicted by arrow (c).

Likewise, from a prevention point of view, it would be advisable to act on various deleterious aspects of the Western lifestyle at a time (diet, food additives, overuse of antibiotics, etc.), with a sustainable long-term objective of avoiding the danger zone and preventing critical transition to a pre-disease state.

## Conclusions

Recent insights in human gut biology indicate the existence of alternative stable states of the gut microbiota–host symbiosis representing health, pre-disease, or different diseases. Each of these states is characterized by microbiota composition and accompanying (alterations in) host physiology and immunity, which are intimately intertwined. The profoundness of the microbiota–host relationship indicates that humans should be considered as holobionts [16, 17] (some authors prefer the term “symbiome” [42]) and, consequently, treatment or prevention of disease should be in line with this view to be successful. Prevention should be aimed at avoiding transition from a healthy state to a pre-disease state, with a key role for nutrition. A combined therapeutic approach, addressing host processes and microbiota, should be used to treat disease.
